# Pulmonary Outcomes in Children Born Extremely and Very Preterm at 11 Years of Age

**DOI:** 10.3389/fped.2021.635503

**Published:** 2021-05-25

**Authors:** Paola Di Filippo, Cosimo Giannini, Marina Attanasi, Giulia Dodi, Alessandra Scaparrotta, Marianna Immacolata Petrosino, Sabrina Di Pillo, Francesco Chiarelli

**Affiliations:** ^1^Department of Pediatrics, University of Chieti, Chieti, Italy; ^2^Pediatric Allergy and Respiratory Unit, Department of Pediatrics, University of Chieti, Chieti, Italy

**Keywords:** prematurity, bronchopulmonar dysplasia, diffusing capacity for carbon monoxide, diffusing capacity for lung carbon monoxide, preschool wheezing

## Abstract

**Background:** There is increasing evidence of prematurity being a risk factor for long-term respiratory outcomes regardless the presence of bronchopulmonary dysplasia (BPD).

**Aim:** To assess the effect of prematurity on respiratory outcomes in children born ≤32 weeks of gestational age at 11 years of age.

**Materials and Methods:** Fifty five ex-preterm children (≤ 32 weeks of gestational age), born in Chieti between January 1, 2006 and December 31, 2007, performed lung function and diffusing capacity test (DLCO) at 11 years of age. Furthermore, allergy evaluation by skin prick test (SPT), eosinophil blood count and assessment of eosinophilic airways inflammation by exhaled nitric oxide (FeNO) were performed. The ex-preterm group was compared to an age- and sex-matched group of term children.

**Results:** No difference for atopic and respiratory medical history was found between ex-preterm children and term controls, except for preschool wheezing that resulted more frequent in ex-preterm children. No difference neither in school-aged asthma frequency nor in lung function assessment at 11 years of age was found between the two groups. Lower DLCO values in ex-preterm children compared to term controls regardless the presence of BPD were found; furthermore, we showed a positive association between DLCO and gestational age. Eosinophil blood count, positive SPTs and FeNO values were similar between the two groups.

**Conclusions:** Diffusing lung capacity was decreased in ex-preterm children at 11 years of age in the absence of lung function impairment and eosinophil airway inflammation, suggesting a non-eosinophilic pattern underlying pulmonary alterations. It could be desirable to include the diffusing capacity assessment in follow-up evaluation of all ex-preterm children.

## Introduction

Neonatal intensive care reached extraordinary improvements during the last three decades. Antenatal corticosteroids in high risk pregnancies, postnatal surfactant replacement therapy and implementation of noninvasive ventilation techniques drastically reduced neonatal mortality and morbidity (1, 2).

However, a large proportion of children born preterm still develop long-term respiratory complications (3). Although pulmonary morbidity often improves during growth, in the majority of children with bronchopulmonary dysplasia (BPD), a subset of children still reports respiratory symptoms during childhood and adolescence (4). These complications affect not only subjects with BPD but even ex-preterm children without BPD (3).

The improved survival rate in infants born at very early gestational ages and a better management of neonatal respiratory distress led to a new pattern of lung injury. “*Old*” BPD was characterized by inflammation, airway smooth muscle hypertrophy, emphysema and parenchymal fibrosis due to high oxygen concentration and high ventilation pressures. The main cause of “*new*” BPD is the alveolar development arrest, which leads to reparative processes, impaired alveolarization with fewer and dysmorphic capillaries, despite a less evidence of emphysema, fibrosis and airway changes (5, 6). This new entity affects only infants born very preterm (1, 3) and it is still not clear whether the *new BPD* is associated with different long-term respiratory outcomes (3).

To date, most studies on long-term lung function focused on outcomes in BPD patients, while there is increasing evidence of prematurity *per se* being a risk factor for respiratory problems, even without BPD. Furthermore, efforts to compare the long-term respiratory outcomes of ex-preterm children with and without BPD are affected by relevant difficulties, and particularly the lack of shared definition (6) and of homogeneous populations.

The aim of the study was to assess the effect of prematurity on respiratory outcomes in children born ≤32 weeks of gestational age compared to full-term controls at 11 years of age. Secondary aims were: (1) to evaluate the frequency of the respiratory symptoms in ex-preterm children; (2) to verify the role of the atopy and eosinophilic airway inflammation in their lung function.

## Materials and Methods

### Study Population

**Figure 1:**
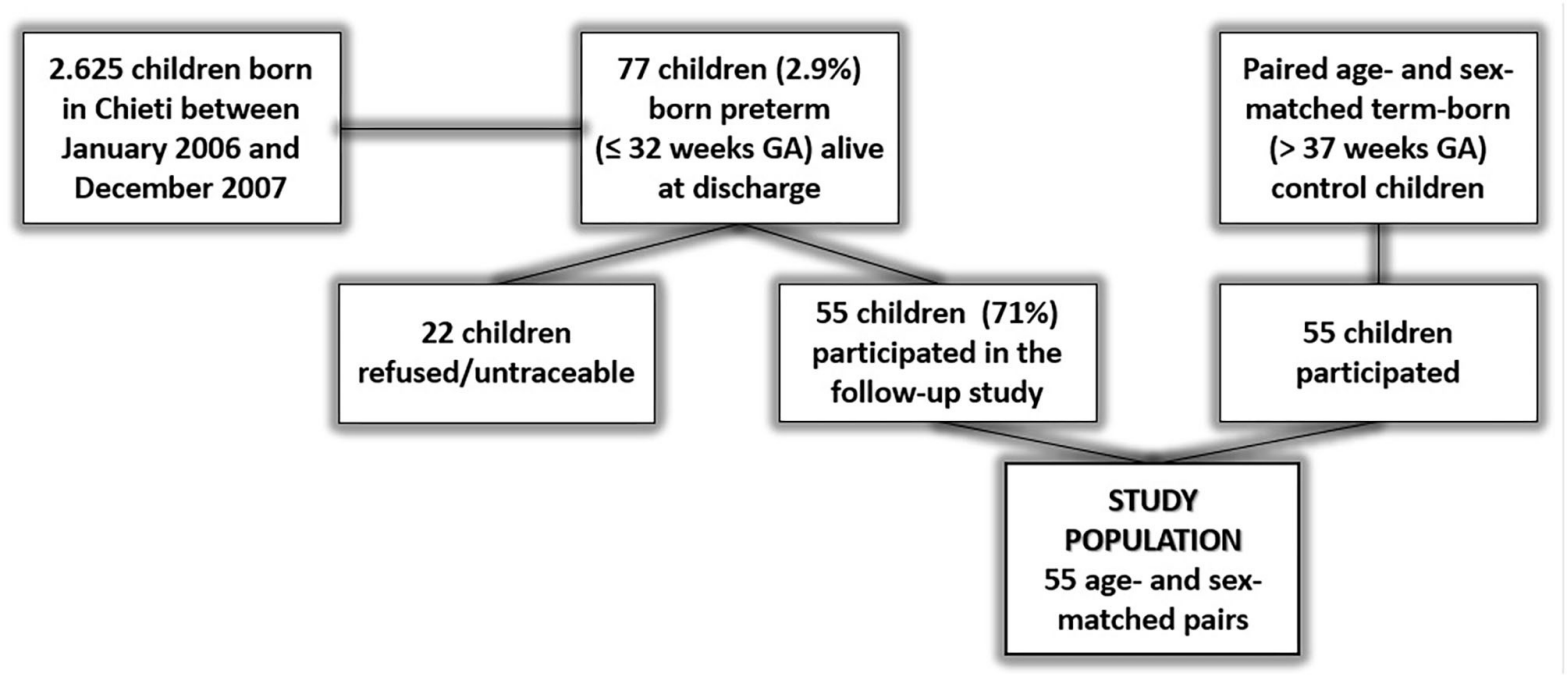
Flow-chart of the study. Children born in Chieti from January 1, 2006 to December 31, 2007 were 2,625. In the same period, infants born ≤32 weeks of gestational age and alive at discharge were 77 (2.9%). Of these 77 children, 55 accepted to be enrolled in the study; 22 refused or were untraceable.

The study was performed at the Pediatric Allergy and Respiratory Center, University Hospital of Chieti, Italy. The study population included all survived ex-preterm children born ≤32 weeks of gestational age between January 1, 2006 and December 31, 2007 and admitted to the Neonatal Intensive Care Unit of Chieti (Figure 1). Perinatal and neonatal information were assessed by consulting their medical records.

School-aged ex-preterm children were asked to participate to our cross-sectional study between May 2017 and May 2018. Children with severe mental disability and/or tetraplegia were excluded. Asthma and atopy were not exclusion criteria.

The diagnosis of BPD was based on the oxygen need for 28 days and additional oxygen or ventilation requirements at 36 weeks' postmenstrual age (6).

An age- and sex-matched group of classmate for each ex-preterm child was sought. Classmates were excluded if they were born earlier than <37 weeks of gestational age. The presence of asthma and atopy was not an exclusion criterion. Controls recruitment was continued until 1:1 ratio was reached.

An accurate medical personal and family history was collected by a pediatric pulmonologist at follow-up visit. Current asthma was defined as physician-diagnosed asthma, obtained by parental questionnaire at age of 11 years, with a positive history of either wheezing or medication in the past 12 months. Preschool wheezing and school-aged asthma were defined as physician-asthma diagnosed until 5 years and from 5 to 10 years of age respectively.

A clinical evaluation including the anthropometric parameters (height, weight, BMI) and pubertal stage was performed at follow-up visit. A venous blood sampling for the eosinophil count, skin prick test for the allergic status, flow/volume curve and plethysmography for the lung function, fractional exhaled nitric oxide (FeNO) measurement for the airway inflammation were obtained at 11 years of age.

Written consent was taken from parents of the subjects. The follow-up study was approved by the Ethical Committee of University of Chieti.

### Measurement of Lung Function

At the follow-up visit, all participants were in stable clinical condition without any acute respiratory symptoms in the previous 2 weeks.

Lung function was assessed in the Pediatric Allergy and Respiratory Clinic of the Department of Pediatrics of Chieti by flow/volume curves (using spirometer Master Screen; Viasys GmbH—Erich Jaeger, Hoechberg, Germany). They were made in the standing position using a nose clip. Until three consecutive technically acceptable curves were achieved according to ATS/ERS guidelines (7). The main spirometric parameters included were: forced expiratory volume in 1 second (FEV_1_), forced vital capacity (FVC), the ratio FEV_1_/FVC, forced expiratory flows between 25 and 75% of the FVC (FEF_75_, FEF_25−75_). Total Lung Capacity (TLC), specific airways resistances (sRaw) and residual volume (RV) were measured using standardized body plethysmography (Vmax® Autobox V62J, Carefusion, Hoechberg, Germany).

Lung function evaluation was performed at least 3 times for each patient; the best spirometric measurements were considered for statistical calculations. The maximal tolerated variability for the three lung function measurements was <10%, as referred by Pellegrino et al. (8).

Bronchodilator test was carried out 15 min after the administration of salbutamol (200 mcg) using a spacer. An increase of at least 12% in FEV_1_ was considered significant for bronchial reversibility (8).

DLCO was measured with a standardized single breath technique (Vmax® Autobox V62J, Carefusion, Hoechberg, Germany); nobody had a history of anemia. After the child established a stable breathing pattern he/she was instructed to exhale completely. The valve to the test gas was then opened and the patient took a full breath. The maneuver was considered reliable if the inspiratory time was <4 s and the inhaled volume was at least 85% of the largest vital capacity. The patient then held his breath for 10 ± 2 s, maintaining near atmospheric pressure during the breath hold (performing neither a Valsalva nor Muller maneuver). DLCO maneuvers were repeated until at least two technically acceptable and reproducible tests were obtained (9).

### Respiratory Inflammation Marker

FeNO was assessed with an on-line method using a single breath exhalation and a sensitive chemiluminescence assay (Ecomedics CLD 88), according to ATS-ERS (10). Patients performed an inspiration of eNO-free air via a mouthpiece immediately followed by full exhalation at a constant rate (50 ml/s) for at least 5 s. The mean of three readings at the end of the expiration (plateau phase) was taken as the representative value for each measurement.

### Assessment of Allergy

Allergic status was assessed by SPT for the most common food and respiratory allergens (Egg white and yolk, Milk, Cod, Tomato, Wheat, Peanut, Shrimp, Parietaria, Grass, Olive, Cypress, Absinthe, Ambrosia, Dermatophagoides pteronyssinus and farinae, Cat and Dog dander, Alternaria alternate). Histamine (10 mg/ml) and saline were used as positive and negative controls respectively; diameters ≥3 mm were considered positive (11).

## Statistical Analysis

Continuous data were presented as mean and standard deviation. Categorical data were presented as numbers and percentages.

Z-scores for DLCO, FEV_1_, FVC, FEF_75_, FEF_25−75_ and FEV_1_/FVC were derived using prediction equations from the Global Lung Function Initiative (GLI-2012) (12, 13) using specialized software (14). The lower limit of normal (LLN) was considered at the 5th percentile of the z-score distribution (13) which corresponded to −1.64. Data on TLC, RV, sRaw were expressed as percentages of predicted for age, height, sex and ethnicity based on the Global Lungs Initiative (GLI) 2012 reference values (13).

Comparisons between ex-preterm children and term controls were performed by *Mann Whitney U-test* for continuous variables and *Chi-squared test* for categorical variables. In order to better characterize the independent effect of prematurity on the pulmonary function, ex-preterm participants were also divided according to the presence of BPD (ex-preterms with BPD and ex-preterms without BPD). *Mann Whitney U-test* was used to evaluate the differences between all ex-preterms and term controls, and between ex-preterms without BPD and term controls. *Spearman correlation* was performed to investigate the relationship between the main variables of interest and DLCO. A Backward linear regression analysis was performed using DLCO z-score as dependent variable and the main variables of interest (Gestational age, BMI, gender, birth weight, CPAP duration, mechanical ventilation duration, breastfeeding) as independent variables.

The statistical significance level was *P* < 0.05. Statistical analysis was performed using SPSS version 20.0.

## Results

Children born in Chieti from January 1, 2006 to December 31, 2007 were 2,625. In the same period, infants born ≤32 weeks of gestational age and alive at discharge were 77 (2.9%). Of these 77 children, 55 accepted to be enrolled in the study; 22 refused or were untraceable. All children were Caucasian. The flow-chart of the study is shown in the Figure 1. The main neonatal parameters were similar between preterm infants included in the study population and preterm infants not enrolled (Supplementary Material).

Neonatal and clinical characteristics of the study population in the previous 11 years of life are shown in Table 1. In terms of prenatal and postnatal risk factors (family history of asthma or allergy, *in utero* smoke exposure, passive smoke, pet keeping, time of weaning) no difference was found between ex-preterm children and term controls. Atopic and respiratory medical history (atopic dermatitis, food allergy, bronchiolitis, allergic rhinitis, pneumonia, urticaria) were similar between ex- preterm children and term controls, except for preschool wheezing prevalence. In fact, ex-preterm children presented more frequently pre-school wheezing compared to term controls (27.3 vs. 7.3%; *P* = 0.01). No difference was found for school aged and current asthma.

**Table 1:** Main clinical characteristics of the two groups of the study population at birth and during the first 11 years of life.

	**All preterms**	**Term controls**	***P*-values**
	**(*N* = 55)**	**(*N* = 55)**	
Sex (M/F)	27/28	27/28	
**Neonatal characteristics**			
Mother age at birth (years)	30.8 ± 5.4	31.3 ± 4.7	
Gestational age (weeks)	30.6 ± 1.6	38.4 ± 1.4	
Cesarean section (Yes/No; %)	54/1 (98.2%)	18/37 (32.7%)	
Birth weight (kg)	1.43 ± 0.4	3.12 ± 0.6	
Birth length (cm)	41.6 ± 2.7	49.6 ± 2.1	
Mechanical Ventilation (days)	1.2 ± 2.5		
CPAP (days)	5.3 ± 6.8		
Oxygen supplementation (days)	7.6 ± 9.6		
Hospital stay (days)	45.5 ± 19.6	4.1 ± 5.8	
**Prenatal and postnatal risk factors**			
Family history of asthma (Yes/No; %)	11/44 (20.0%)	12/43 (21.8%)	0.86
Family history of allergy (Yes/No; %)	19/36 (34.5%)	18/37 (32.7%)	0.84
*In utero* smoke exposure (Yes/No; %)	7/48 (12.7%)	5/50 (9.1%)	0.76
Passive smoking (Yes/No; %)	23/32 (41.8%)	18/37 (32.7%)	0.43
Pet keeping (Yes/No; %)	28/27 (50.9%)	20/35 (36.4%)	0.18
Breastfeeding (months)	4.3 ± 5.3	10.2 ± 6.9	**<0.001**
**Medical history**			
Atopic dermatitis (Yes/No; %)	8/47 (14.5%)	12/43 (21.8%)	0.46
Food allergy (Yes/No; %)	6/49 (10.9%)	4/51 (7.3%)	0.74
Bronchiolitis (Yes/No; %)	7/48 (12.7%)	5/50 (9.1%)	0.76
Allergic Rhinitis (Yes/No; %)	19/36 (34.5%)	23/32 (41.8%)	0.56
Pneumonia (Yes/No; %)	5/50 (9.1%)	6/49 (10.9%)	0.76
Urticaria (Yes/No; %)	6/49 (10.9%)	9/46 (16.4%)	0.58
Preschool wheezing (Yes/No; %)	15/40 (27.3%)	4/51 (7.3%)	**0.01**
School aged asthma (Yes/No; %)	6/49 (10.9%)	4/51 (7.3%)	0.74
Current asthma (Yes/No; %)	3/52 (5.5%)	4/51 (7.3%)	0.86

*Values are absolute numbers (percentages) for categorical data and mean ± standard deviation for continuous variables*.

*N, numbers; CPAP, continuous positive airway pressure*.

*The statistical significance level is p < 0.05 and it is indicated in bold*.

The anthropometric, atopic and lung function data at 11 years of age are presented in Table 2. At the follow-up visit, all participants were pre-pubertal.

**Table 2:** Anthropometric, atopic, and lung function data at 11 years of the study population.

	**Term controls (*N* = 55)**	**All preterms (*n* = 55)**	**PRETERMS NO BPD (*N* = 50)**	**Preterms with BPD (*N* = 5)**	***P*-values terms vs. preterms**	***P*-values BPD vs. no BPD**	***P*-values terms vs. no BPD**
**Anthropometric data**							
Age (years)	11.1 ± 0.9	11.0 ± 0.6	11.0 ± 0.6	11.1 ± 0.8	0.81	0.86	0.77
Weight (kg)	43.3 ± 9.7	42.3 ± 10.0	42.4 ± 9.9	41.6 ± 11.9	0.64	0.82	0.81
Height (cm)	148.2 ± 10.7	145.3 ± 8.1	145.3 ± 8.3	145.2 ± 6.6	0.49	0.95	0.50
BMI (kg/m^2^)	19.6 ± 3.1	19.8 ± 3.4	19.8 ± 3.4	19.5 ± 3.9	0.71	0.77	0.65
**Atopic characteristics**							
EO%	3.1 ± 2.0	3.5 ± 2.5	3.2 ± 2.2	5.1 ± 3.7	0.61	0.36	0.86
SPT positive	32.7%	38.2%	36.0%	60.0%	0.69	0.35	0.83
FeNO (ppb)	10.8 ± 4.1	10.7 ± 5.3	10.4 ± 4.8	14.6 ± 9.5	0.45	0.31	0.39
**Lung function**							
FEV_1_ Z-score	0.7 ± 0.9	0.5 ± 1.3	0.6 ± 1.3	0.1 ± 1.1	0.49	0.34	0.74
FVC Z-score	0.2 ± 0.6	0.2 ± 1.2	0.2 ± 1.2	−0.1 ± 1.1	0.94	0.41	0.91
FEV_1_/FVC Z-score	0.7 ± 0.9	0.6 ± 1.0	0.6 ± 1.1	0.4 ± 0.6	0.72	0.62	0.79
FEF_75_ Z-score	1.3 ± 0.8	1.2 ± 1.1	1.3 ± 1.0	0.6 ± 0.5	0.33	0.09	0.82
FEF_25−75_ Z-score	0.5 ± 0.7	0.3 ± 0.9	0.3 ± 0.9	−0.1 ± 0.5	0.16	0.16	0.31
sRaw%	169.2 ± 24.9	187.7 ± 48.1	184.4 ± 48.9	219.6 ± 23.4	**0.04**	**0.02**	0.16
VR%	106.9 ± 32.6	105.9 ± 54.4	106.2 ± 56.9	103.2 ± 23.3	0.22	0.48	0.19
TLC%	103.9 ± 16.1	98.5 ± 14.9	98.5 ± 15.6	98.4 ± 4.0	**0.02**	0.33	**0.01**
Bronchoreversibility	3.6%	9.1%	8.0%	20.0%	0.44	0.39	0.42

*Values are percentages for categorical data and mean ± standard deviation for continuous variables*.

*BPD, bronchopulmonary dysplasia; BMI, body mass index; EO%, serum eosinophils; SPT, skin prick test; FeNO, fractional exhaled nitric oxide; DLCO, carbon monoxide diffusing capacity; FEV_1_, forced expiratory volume in the 1st second; FVC, forced vital capacity; FEF_75_, forced expiratory flow at 75% of FVC; FEF_25−75_, forced expiratory flow between 25 and 75% of FVC; sRaw, specific airway resistance; VR, residual volume; TLC, total lung capacity*.

*The statistical significance level is p < 0.05 and it is indicated in bold*.

**Figure 2:**
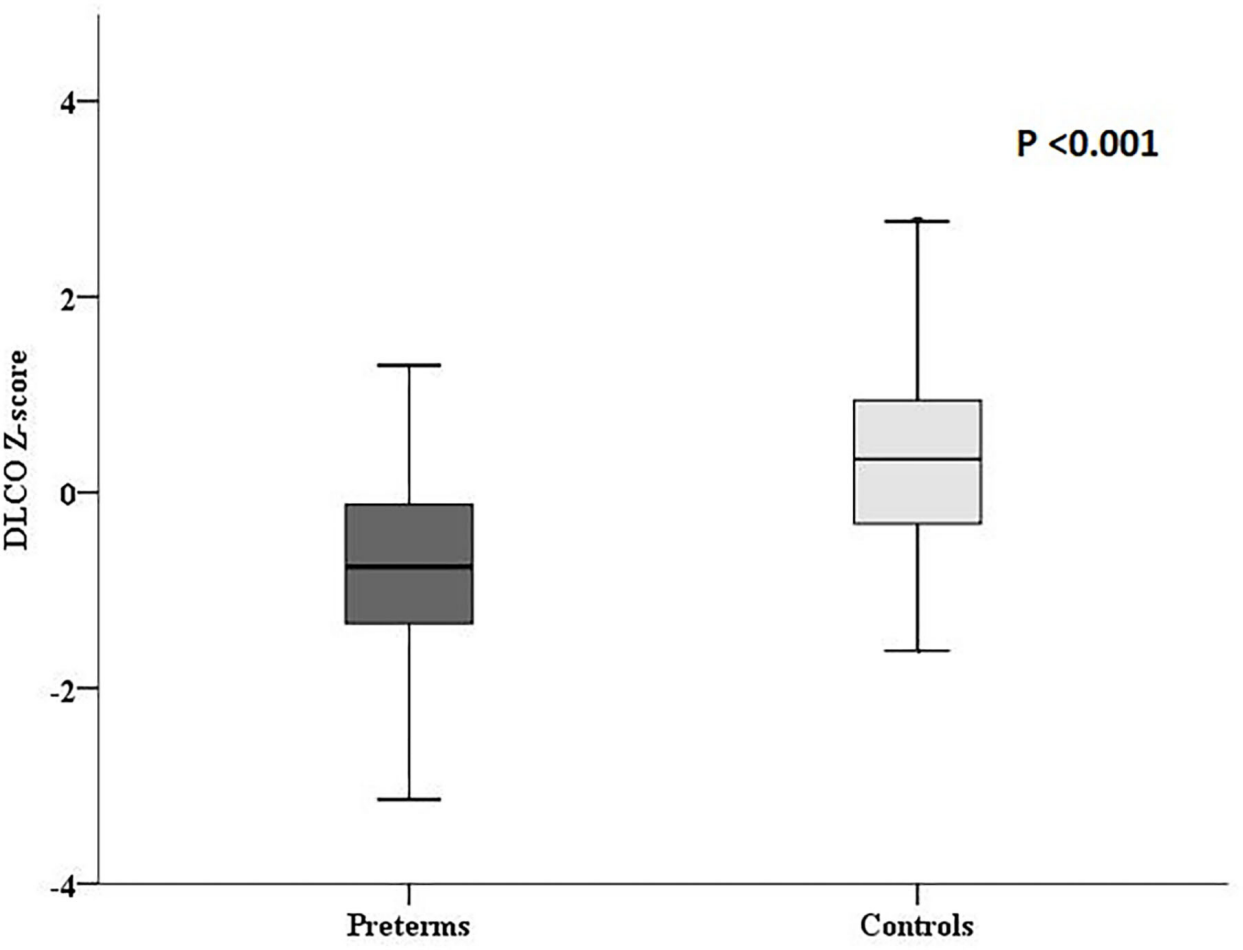
DLCO z-scores in ex-preterm children and term controls at 11 years of age.

**Figure 3:**
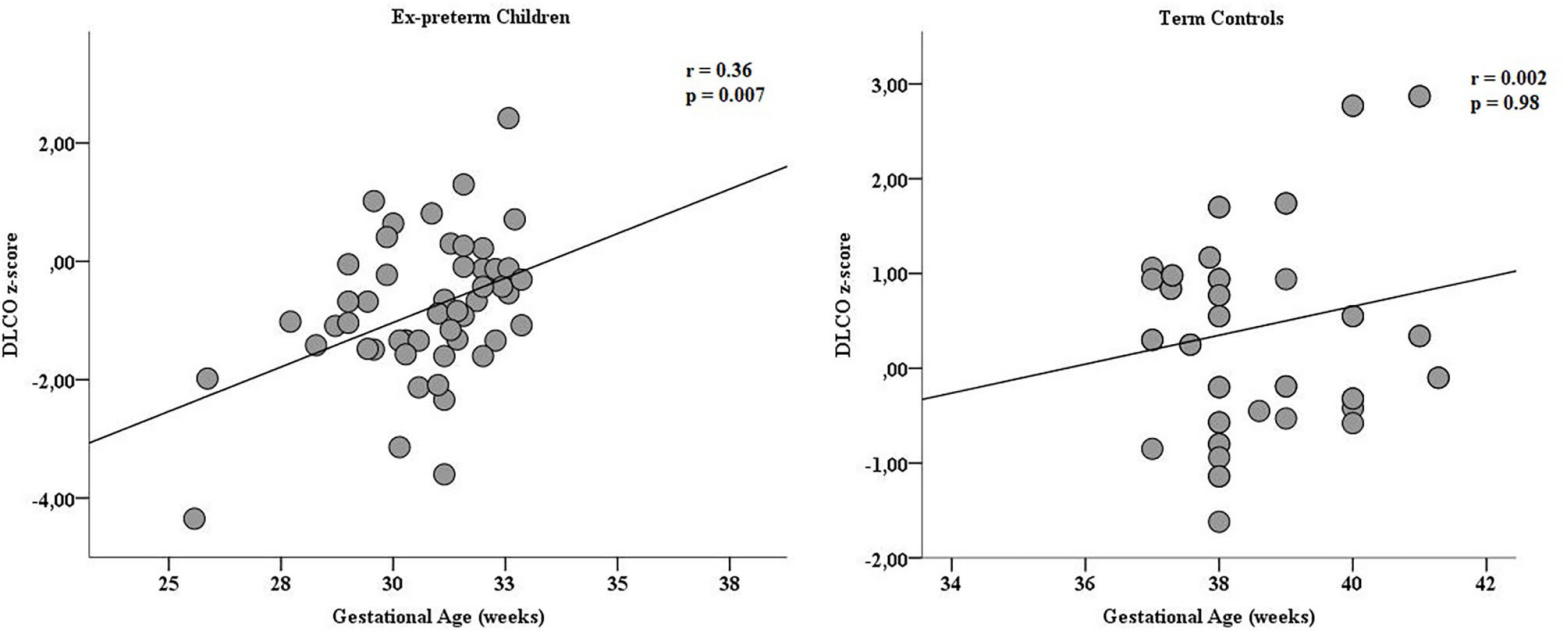
Correlation of gestational age (expressed in weeks) and DLCO z-score in preterm children and term controls at 11 years of age. The positive correlation was found only in the preterm group.

DLCO z-score was significantly lower in ex-preterm children than term controls (−0.8 ± 1.2 vs. 0.4 ± 1.1, *P* < 0.001; Figure 2); noteworthy, we found a significant difference in DLCO between ex-preterm children without BPD and term children (−0.6 ± 1.1 vs. 0.4 ± 1.1, *P* < 0.001). A positive correlation between gestational age and DLCO z-score values was documented in ex-preterm children (Figure 3). The linear regression analysis confirmed the association between gestational age and DLCO z-score (Table 3).

**Table 3:** Aassociation between the main variables of interest and DLCO by a multi stepwise linear regression analysis.

	**Regression coefficient**	***P*-value**
Gestational age	0.52	**0.000**
Birth weight (kg)	0.07	0.76
Sex	0.13	0.14
Mechanical ventilation duration (days)	0.11	0.25
CPAP duration (days)	0.17	0.12
Breastfeeding duration (months)	0.13	0.16
Body mass index at follow-up visit (kg/m^2^)	0.14	0.12

*CPAP, continuous positive airway pressure*.

*The statistical significance level is p < 0.05 and it is indicated in bold*.

Among ex-preterm children, 5 (9.1%) had BPD (Table 2). Ex-preterm children with BPD showed worse specific airways resistances values and lower DLCO z-score values compared to ex-preterm children without BPD (sRaw%: 219.6 ± 23.4 vs. 184.4 ± 48.9, *P* = 0.02; DLCO z-scores −1.7 ± 0.8 vs. −0.6 ± 1.1, *P* = 0.04, respectively).

We also found lower DLCO z-score values in ex-preterm without BPD compared to term controls (−0.7 ± 1.2 vs. 0.4 ± 1.1, *P* < 0.001), thus highlighting the effect of prematurity on alveolar-capillary membrane also without the presence of BPD.

Lung function expressed by flow/volume parameters (FEV_1_ z-score, FVC z-score, FEV_1_/FVC z-score, FEF_75_ z-score, FEF_25−75_ z-score) was not significantly different between the two groups. Bronchodilator reversibility was not significantly different between the two groups. At plethysmography, sRaw% and TLC% were significantly different between ex-preterms and term controls (187.7 ± 48.1 vs. 169.2 ± 24.9, *P* = 0.04 and 98.5 ± 14.9 vs. 103.9 ± 16.1, *P* = 0.02, respectively).

No difference in term of atopic characteristics (serum eosinophils, skin prick test and FeNO) were found between the ex-preterm children and term controls (Table 3).

## Discussion

In our study ex-preterm children reported higher frequency of preschool wheezing than term controls. These results are also confirmed in several studies (15–17) and in a large meta-analysis with 1.5 million children from six continents finding that prematurity was associated with a 1.7-fold higher risk of childhood wheezing disorders (18).

In the past, the higher frequency of wheezing in all subjects born prematurely was interpreted as asthma. To date the mechanisms of airflow obstruction in subjects with a history of prematurity are not entirely understood. Kennedy et al. (15) proposed that fewer alveoli and alveolar attachments in preterm lung causes more difficult opening of small airways during a period of rapid postnatal lung growth. This finding leads to an impaired airway growth and fixed airflow obstruction similar to Chronic Obstructive Pulmonary disease (COPD).

We found a normal lung function in ex-preterm children compared to controls at 11 years of age despite higher frequency of preschool wheezing. Scientific evidence is still conflicting regarding the persistence of impaired lung function in ex-preterm children. On one hand, a lot of studies showed lung function impairment in preterm children (19–21). On the other hand, several studies observed inconsistent association between birth weight and FEV1/FVC (22, 23). Kitchen et al. (24) found that lung function was similar in very low birth weight children compared to normal weight birth ones at 8 years of age, despite very low birth weight children suffered more frequently from wheezing in the first 2 years of life. In a recent study performed in preschool aged children, a similar lung function in mild BPD ex-preterm subjects compared to ex-preterm ones without BPD and term controls has been shown, suggesting that mild BPD might not lead to long-term respiratory consequences (21). The available conflicting findings might be partially explained by the small study sample size, the heterogeneity in the definition of BPD and in the lung function methods utilized, and the different age in which lung function was assessed in cross-sectional studies. Few longitudinal studies were performed. A recent longitudinal study assessing lung function at different time points (6, 12, 18, and 24 months) found that lung function in preterm infants with mild to moderate BPD improved gradually (25).

To date several studies include adults with “old BPD” born before surfactant era and very few studies have evaluated lung function of ex-preterm adults with “new BPD.” New interesting insights have been offered by a recent study including young adults born prematurely between 1987 and 1998. In this study authors found lower z- score FEV_1_/FVC, FEV_1_, DLCO and lung density (quantified by CT scanning) in subjects with BPD than those without BPD. These parameters were above the lower limit of normal in 55% of recruited subjects, suggesting recovery of lung function impairment in a reasonable percentage of survivors of BPD (26).

These recent findings suggest that ex-preterm children with early reduced lung function compared to term controls could improve lung function over time, especially in absence of BPD (15, 25, 27). According to these findings, we speculate that the lung function was normal in our study population at 11 years of age probably because their lung function improved over time. However, higher percentage of ex-preterm children without BPD than ones with BPD could influence mean values of the lung function parameters.

Noteworthy, we showed that children born ≤32 weeks of gestational age presented an impaired diffusing capacity compared to full-term controls at 11 years of age. Lower DLCO z-score values persisted in ex-preterm subjects without BPD compared to term controls, highlighting the effect of prematurity on alveolar-capillary membrane also without BPD.

DLCO is a compound measure, reflecting lung volume, surface area accessible for gas exchange, thickness of the alveolar capillary barrier and pulmonary capillary blood volume (28). We expected lower DLCO values in ex-preterm children than controls given that the alveolar-capillary membrane grows considerably between 22 and 32 weeks of gestational age (5). The disruption in alveolar and vascular growth in preterm subjects result in more simplified pulmonary acini and vascular structures (5), reducing the surface area for gas exchange, as confirmed in autopsy findings in preterm infants (29). The overall reduction in number of pulmonary capillaries with the remaining vessels being dysmorphic and far from the airspace surface result in thickened alveolar-capillary membrane (30).

Several studies found that DLCO is reduced by about 10% in subjects born preterm compared with subjects born at term (28, 31, 32); particularly, two studies evaluating extremely preterm children demonstrated ventilation inhomogeneity and gas transfer impairment in ex-preterm subjects at 11 years of age (32, 33).

Importantly, we showed a positive association between gestational age and DLCO in ex-preterm children, which persisted after adjusting for birth weight, CPAP duration, mechanical ventilation duration, breastfeeding, BMI and sex, suggesting an independent effect of prematurity on DLCO values. According to these findings, both Hakulinen et al. (31) and Um-Bergström et al. (34) demonstrated a disturbance of gas diffusion in presence of a normal lung function in ex-preterm young adults without previous diagnosis of BPD. In addition, as also suggested recently, in ex-preterm children the alteration of diffusing capacity at 10 years of age could be the hallmark of an underlying peripheral airway impairment (35).

FeNO values, SPT positivity and eosinophil blood count were similar in ex-preterm group compared to term controls. These findings might suggest that lung diffusing capacity is not induced by eosinophil airways inflammation and that airways inflammation is different in ex-preterm children compared to asthmatics children. A recent meta-analysis with 640 preterms and 4,005 term controls found no difference in FeNO levels between ex-preterm and term subjects and between ex-preterm subjects with and without Chronic Lung Disease (36). To date, several studies have shown no eosinophilic markers in ex preterm children (35, 37) suggesting an alternative inflammation pattern underlying wheezing and airway obstruction in ex-preterms subjects. Indeed BPD seems to be characterized by persistent neutrophilic airway inflammation and oxidative stress resembling inflammatory mechanisms of COPD (4).

The main strength of our study are the simultaneous assessment of lung function, diffusing capacity and airways eosinophilic inflammation providing a comprehensive respiratory evaluation of the participants. Secondly, our study include children born at high-resource tertiary hospital in the post-surfactant era with “*new* BPD” respect to several available studies on school aged children or teenagers including subjects with “*old* BPD.” Furthermore, spirometric and DLCO parameters were expressed as z-score according to the equation GLI-2012 (14), minimizing the different effects of age, sex, height and race. All measurements were performed by the same operator with expertise in lung function methods. Lastly, the presence of a control group supports the reliability of our findings.

The main limitation of our study are the cross-sectional analysis and the lack of lung function assessment in the first years of life when respiratory symptoms were present. However, we collected data about the previous 11 years of life (prenatal, perinatal and postnatal factors). Another important limitation was the small sample size of the study population, in particular of the preterms with BPD resulting in a very skewed comparison between preterm with and without BPD.

In conclusion, we found that ex-preterm children report more frequently preschool wheezing compared to term children. No significant difference in terms of frequency of school aged-asthma and current asthma and in the lung function values between ex-preterm children and term controls at 11 years of age was found, suggesting a potential gradual clinical improvement over time in ex-preterm children. In addition, no significant difference was also found in eosinophilic airways inflammation and atopic characteristics between the two groups. Therefore, we postulate that the pathological mechanism and the underlying airways inflammation of chronic lung disease of prematurity could be inherently different from asthma (38, 39). Importantly, we showed a reduced diffusing capacity in ex-preterm children compared to full-term controls at 11 years of age. Further, longitudinal prospective studies with a larger sample size of “*new* BPD” children and with periodic evaluation of lung function from birth to adulthood are needed to better characterize lung function changes over time. However, our findings highlight the importance to follow-up ex-preterm children with a periodic lung function assessment which includes not only spirometry but also diffusing capacity testes.

## Data Availability Statement

The raw data supporting the conclusions of this article will be made available by the authors, without undue reservation.

## Ethics Statement

This study was approved by the Ethics Committee of the University of Chieti. A written informed consent to participate in this study was provided by the participants' legal guardian/next of kin.

## Author Contributions

PDF contacted and recruited patients at 11 years of age and wrote the manuscript. CG performed the statistical analysis. GD participated in the writing of the manuscript. AS analyzed neonatal records of patients. MIP participated to the recruitment of patients. MA, SDP, and FC revised the manuscript. All authors contributed to the article and approved the submitted version.

## Conflict of Interest

The authors declare that the research was conducted in the absence of any commercial or financial relationships that could be construed as a potential conflict of interest.

## Abbreviations

BPD: bronchopulmonary dysplasia
DLCO: diffusing capacity of the lung for carbon monoxide
SPT: skin prick test
FeNO: fractional exhaled nitric oxide
BMI: body mass index
ATS: American thoracic society
ERS: European respiratory society
FEV_1_, FVC: forced expiratory volume in the first second, forced vital capacity
FEF_25−75_: forced expiratory flow at the 25–75% of the pulmonary volume
TLC: total lung capacity
sRaw: specific airways resistances
RV: residual volume
SD: standard deviation
GLI-2012: global lung function initiative
LLN: lower limit of normal
COPD: chronic obstructive pulmonary disease.
