# Supplementary material for: Pulmonary Outcomes in Children Born Extremely and Very Preterm at 11 Years of Age
Source: Front Pediatr. 2021 May 25;9:635503. doi: 10.3389/fped.2021.635503 (PMC8185052; doi:10.3389/fped.2021.635503)
Supplement: Supplementary file 1 [file Table_1.docx]

**Table 1S.** Main perinatal characteristics between preterms enrolled and preterms not enrolled.

|  | Preterms enrolled  (N =55) | Preterms not enrolled  (N=22) | *p*  *value* |
| --- | --- | --- | --- |
| Sex (M/F) | 27/28 | 13/9 | 0.67 |
| Mother age at birth (years) | 31.4±4.8 | 31.3±5.3 | 0.53 |
| Twins (Yes/No; %) | 19/36 (34.5%) | 15/7 (68.2%) | **0.01** |
| Gestational age | 30.7±1.7 | 31.0±2.1 | 0.15 |
| Caesarean section (Yes/No; %) | 54/1 (98.2%) | 20/2 (90.9%) | 0.19 |
| Cephalic presentation (Yes/No; %) | 33/22 (60.0%) | 13/9 (59.1%) | 0.80 |
| Apgar 1' | 6.5±1.4 | 6.3±1.7 | 0.83 |
| Apgar 5' | 8.0±0.8 | 7.8±1.5 | 0.82 |
| Cord pH | 7.4±0.1 | 7.3±0.1 | 0.14 |
| Cord BE | -3.5±3.3 | -4.1±4.6 | 0.96 |
| Birth weight (kg) | 1.4±0.4 | 1.49±0.4 | 0.47 |
| Birth length (cm) | 41.3±2.8 | 40.9±2.9 | 0.82 |
| Cranial circumference (cm) | 29.3±2.3 | 29.2±2.5 | 0.67 |
| Mechanical ventilation (days) | 1.2± 2.6 | 2.1±4.2 | 0.85 |
| CPAP (days) | 5.3±6.9 | 3.9±7.7 | 0.32 |
| Oxygen supplementation (days) | 7.6±9.6 | 5.2± 8.9 | 0.27 |
| Hospital stay (days) | 45.5±19.7 | 42.3±17.8 | 0.61 |
| Antenatal steroids (Yes/No; %) | 36/19 (65.5%) | 13/9 (59.1%) | 0.61 |
| Exogenous surfactant (Yes/No; %) | 47/8 (85.5%) | 20/2 (90.9%) | 0.71 |
| Caffeine (Yes/No; %) | 49/6 (89.1%) | 15/7 (68.2%) | **0.04** |
|  |  |  |  |

*Values are absolute numbers (percentages) for categorical data and mean ± standard deviation for continuous variables.*

***N****: numbers;* ***Cord BE****= Cord Base Excess;* ***CPAP****= Continuous Positive Airway Pressure.*
